# Barriers to and Facilitators of Research Utilization: A Survey of Registered Nurses in China

**DOI:** 10.1371/journal.pone.0081908

**Published:** 2013-11-29

**Authors:** Li-Ping Wang, Xiao-Lian Jiang, Lei Wang, Guo-Rong Wang, Yang-Jing Bai

**Affiliations:** 1 West China School of Nursing, West China School of Medicine/West China Hospital, Sichuan University, Wu Hou District, Chengdu, Sichuan Province, P. R. China; 2 Nursing Department of The Second People’s Hospital of Sichuan Province, Wu Hou District, Chengdu, Sichuan Province, P. R. China; University of Maryland, School of Medicine, United States of America

## Abstract

**Aims:**

This survey aims to describe the perception of barriers to and facilitators of research utilization by registered nurses in Sichuan province, China, and to explore the factors influencing the perceptions of the barriers to and facilitators of research utilization.

**Methods:**

A cross sectional survey design and a double cluster sampling method were adopted. A total of 590 registered nurses from 3 tertiary level hospitals in Sichuan province, China, were recruited in a period from September 2006 to January 2007. A modified BARRUERS Scale and a Facilitators Scale were used. Data were analyzed with descriptive statistics, rank transformation test, and multiple linear regression.

**Results:**

Barriers related to the setting subscale were more influential than barriers related to other subscales. The lack of authority was ranked as the top greatest barrier (15.7%), followed by the lack of time (13.4%) and language barrier (15.0%). Additional barriers identified were the reluctance of patients to research utilization, the lack of funding, and the lack of legal protection. The top three greatest facilitators were enhancing managerial support (36.9%), advancing education to increase knowledge base (21.1%), and increasing time for reviewing and implementing (17.5%), while cooperation of patients to research utilization, establishing a panel to evaluate researches, and funding were listed as additional facilitators. Hospital, educational background, research experience, and knowledge on evidence-based nursing were the factors influencing perceptions of the barriers and facilitators.

**Conclusions:**

Nurses in China are facing a number of significant barriers in research utilization. Enhancing managerial support might be the most promising facilitator, given Chinese traditional culture and existing health care system. Hospital, educational background, research experience and knowledge on evidence-based nursing should be taken into account to promote research utilization. The BARRIERS Scale should consider funding and involvement of patients in research utilization.

## Introduction

In pursuit of professionalism and quality care, utilization of research results in practice has been considered vital to nursing profession. The widely recognized evidence-based movement has put more emphasis on the importance of research utilization. To improve clinical cost-effectiveness and outcomes, professional associations and educators have required nurses to build their practice on research findings [1,2]. However, despite the increasing quantity and improving quality of nursing researches, incorporating research results into clinical practice remains a significant challenge [3-7]. 

### Barriers to research utilization

Hunt suggested that barriers to research utilization cover five domains: the quality of research, the access to research, the process of research utilization, the attitude and knowledge of nurses, and the organization into which research results are to be applied [8]. Kitson conceptualized barriers to research utilization as three interactional types: the level and nature of the evidence, the context or environment into which the research is to be implemented, and the method or way in which the implementation is facilitated. Barriers related to the level and nature of evidence include poor quality of research, inconsistent research results, and no participation of patients in research. Barriers related to the context or environment involve task-driven organizational culture, low regard for individuals, little or no continuing education, diffuse roles, lack of team roles, poor leadership, and absence of appropriate performance measurement. Barriers related to the facilitating method or way consist of inflexible style, inappropriate characteristics, and inappropriate role [9]. According to Rich MW, barriers to research utilization could be summarized into three general categories: health professional-related, patient-related, and health system–related. Health professional-related barriers include lack of knowledge of research findings, time constraints, and avoidance of risk. Patient-related barriers mostly lie in difficulties engaging in health-modifying behaviors. Health system-related barriers are mainly about funding [10]. To enhance effectiveness of clinical practice, the Cochrane Effective Practice and Organization of Care Group (EPOC) proposed a classification system to address barriers to research utilization. It includes eight categories: evidence and clinical uncertainty, perception of competence, perception of responsibility, expectation of patient, standards of practice, financial limitation, administrative constraints, and others [11]. 

Of these barriers studies, a quite influential one was conducted by Funk et al. in 1991 [12], which sorted barriers to research utilization into four types: characteristics of the research, characteristics of the nurse, characteristics of the setting, and characteristics of the communication, and is in accordance with Roger’s model of “diffusion of innovations,” a theoretical framework. According to Funk, characteristics of the research included methodological inadequacies, inappropriate conclusions, not replicated research, and conflicting results. Characteristics of the nurse involved their research values, skills and awareness, such as distrust of research, disregard of research, and lack of knowledge to evaluate quality of research findings. Characteristics of the setting comprised barriers and limitations perceived in the work setting, including lack of authority, lack of time, lack of support, and lack of facilities to translate research findings into practice. Characteristics of the communication meant presentation and accessibility of research, including understandability of statistical analysis, no ready or easy access to research findings, obscurity of research articles, and irrelevance to clinical practice [12]. 

### The BARRIERS Scale

To examine opinions of nurses on barriers to research utilization, Funk developed the BARRIERS Scale based on previous literature, the Conduct and Utilization of Research in Nursing (CURN) Questionnaire, and informal data collected from nurses. Face validity and content validity were established by a group of experts. Exploratory factor analysis was performed on two subgroups independently and the entire sample (1,989 nurses, response rate 40%) to examine construct validity, resulting in four-factor solutions, which were labeled as research (6 items), nurse (8 items), setting (8 items), and presentation (6 items). Reliability was confirmed by internal consistent reliability and test-retest reliability. Cronbach’s alpha values for four subscales were 0.65-0.80 and the test-retest reliability 0.68-0.83 [12]. 

The BSRRIERS Scale has been used widely to address barriers to research utilization since development, especially in the western countries [13-15]. A number of researchers performed exploratory or confirmatory factor analysis to verify the factor model proposed by Funk [16-22]. Similar or identical four-factor solutions were obtained in Australian [16,18], UK [17], Iran [19] and Turkey [20], indicating that the BSRRIERS Scale had a stable construct validity in deferent countries and in deferent language versions. In addition, the BARRIERS Scale was found to be sensitive enough to detect changes in barriers perception [23,24], and to discriminate research users from non-research users in perceptions of barriers [25]. These findings suggested that BARRIERS Scale was a useful organizational diagnostic tool to assess barriers to research utilization, and to evaluate the effect of interventions in reducing barriers to research utilization as well [13,14]. 

Researchers believed that strategies need to be developed according to barriers identified circumstance-specifically to enhance research utilization [10]. Although studies on barriers to research utilization have been carried out thoroughly in western countries, no such a study was conducted in China, in which health system, traditional culture, and nursing educational development level are different from western countries. Besides, China has one of the largest nursing groups in the world [26] and the need to promote research utilization is especially important. To bridge the gap between research and practice in China, we conducted this survey to examine the barriers to and facilitators of research utilization and explore the factors influencing the barriers and facilitators.

## Methods

A cross sectional survey was conducted and a double cluster sampling method was used to examine perceptions of barriers and facilitators in registered nurses from tertiary-level hospitals in Sichuan province, China, from September 2006 to January 2007. A total of 590 registered nurses were recruited randomly and proportionally from 3 hospitals that were randomly selected from 9 local tertiary-level hospitals. The questionnaires were enveloped and handed out to selected nurses in each sampled ward. The designated nurses were responsible for distribution and collection of questionnaires. On the envelope, the purpose of this study, the rights of nurses to decide whether to participate, the anonymity of responses were described. Return of completed questionnaire implied consent to participate in this survey. All data were collected with institutional review board approval from the Ethics Committee of Sichuan University. Permission to use the Chinese version of the BARRIERS Scale and Facilitators Scale was obtained from Professor Thompson and Chau, The Chinese University of Hong Kong.

The instrument included demographics, the modified BARRIERS Scale (31 items), and the Facilitators Scale (8 items). The BARRIERS Scale and the Facilitators Scale were translated into Chinese by Thompson et al., in which the forward translation and backward translation were performed to confirm conceptual equivalence of the items. We made some modifications to the BARRIERS Scale as follows. The item “the nurse is unwilling to try/change new ideas” in the original scale was split into two items: “The nurse is unwilling to change practice” and “The nurse is unwilling to try new ideas” to improve clarity; and “research reports/articles are published in English and therefore difficult to understand” was added because English is not the mother tongue for Chinese people [27]. 

Participants were asked to rate each item of the BARRIERS/Facilitators Scale, with 1= to no extent, 2= to a little extent, 3= no opinion, 4= to a moderate extent, and 5= to a large extent. Moreover, participants were asked to identify and rate items from the BARRIERS/Facilitators Scale they perceived to be the top three greatest barriers/facilitators (1=greatest barrier/facilitator, 2=second greatest barrier/facilitator, and 3=third greatest barrier/facilitator). Further to this, the participants were invited to list any additional barrier/facilitator they perceived in their research utilization beyond the BARRIERS/Facilitators Scale. 

The content validity index of the Chinese version of the BARRIERS Scale was 0.98 [27], and in our pre-survey Cronbach’s alpha values were 0.86 for the Chinese version of the BARRIERS Scale, 0.63-0.79 for the four subscales, and 0.89 for the Facilitators Scale. 

All data were analyzed using Statistical Package Social Sciences (version11.5, SPSS Inc., Chicago, IL, USA). Frequency, percentage, and inter-quartile range were employed in description of variables. Rank transformation test was used to analyze the difference in scores of subscales and nurses with different demographic characteristics due to non-normal distribution. Rank transformation test is a kind of nonparametric test, so we first ranked scores of the questionnaire and then performed one-way ANOVA test to analyze the ranks instead of scores [28]. When difference is statistically significant, Scheffe method was applied for multiple comparisons. With statistically significant variables from univariate analysis as independent variables and scores of subscales as dependent variables, Stepwise method was employed for multiple linear regression. As unordered variables, hospitals were transformed into dummy variables in multiple linear regression with hospital A as reference [29].

## Results

### Description of the participants

Among the 590 questionnaires, 555 were returned (94.07%), in which 34 were excluded due to unanswered items, with the final valid sample size being 521. The gender of participants was predominantly female (99.2%) and medians of age and clinical experience were 27 and 7 years, respectively. The majority of participants held an associate degree (71.6%), had no research experience (83.1%), and possessed no knowledge on evidence-based nursing (60.7%). The demographic characteristics of the participants (Table 1) were consistent with findings from a previous survey of nursing human resource in Sichuan province [30]. 

**Table 1:** Demographic characteristic of participants (N=521).

Variable	N (%)	Max-Min (Median)
Gender		
Male	4(0.8)	
Female	517(99.2)	
Age (years)		19-54(27)
Clinical experience (years)		1-34(7)
Education background		
Diploma	54(10.4)	
Associate	373(71.6)	
Bachelor	94(18.0)	
Research experience		
Yes	88(16.9)	
No	433(83.1)	
Knowledge on Evidence-based nursing		
Yes	205(39.3)	
No	316(60.7)	

### Perceptions of Barriers to Research Utilization

It was found that four subscales (setting, nurse, research and presentation) were statistically significant different in scores (F=47.004, P<0.001). Scores of the setting subscale were the highest and scores of the nurse the lowest, indicating that barriers related to the setting were the most influential in research utilization. The rank order and percentage of items perceived as moderate or great barrier are summarized in Table 2. More than half of the participants ranked 8 items as a moderate or great barrier, of which 6 items were setting-related. Top three greatest barriers were identified to be lack of authority (15.7%), lack of time (13.4%), and the language barrier (15.0%). The scattered frequency of top three greatest barriers suggests that participants perceived many barriers as significant barriers. Interestingly, the top three in rank order of items perceived as moderate or great barrier did not match the top three greatest barriers identified by nurses. Further to the barriers items, participants identified additional barriers, including the reluctance of patients to research utilization (3.64%), lack of funding (2.88%), and lack of legal protection from clinical risk of research utilization (1.0%).

**Table 2:** Barriers items in rank order(N=521).

Rankorder	Barriers items	Subscale	Rating item as moderate or great barrier N(%)
1	The facilities are inadequate for implementation	Setting	366 (70.2)
2	Research reports/articles are published in English therefore difficult to understand	New item	353 (67.8)
3	Physicians will not cooperate with the implementation	Setting	315 (60.5)
4	The nurse does not feel she or he has enough authority to change patient care procedures	Setting	299 (57.4)
5	There is insufficient time on the job to implement new ideas	Setting	293 (56.2)
6	The nurse is isolated from knowledgeable colleagues with whom to discuss the research	Nurse	290 (55.7)
7	The nurse does not have time to read research	Setting	277 (53.2)
8	The nurses feels the results are not generalisable to own setting	Setting	264 (50.7)
9	The research has not been replicated	Research	260 (49.9)
10	The research has methodological inadequacies	Research	259 (49.7)
11	Research reports/articles are not readily available	Presentation	255 (48.9)
12	The nurse does not feel capable of evaluating the quality of the research	Nurse	248 (47.6)
13	The conclusions draw from the research are not justified	Research	238 (45.7)
14	The literature reports conflicting results	Research	225 (43.2)
15	Research reports/articles are not publishes fast enough	Research	224 (43.0)
16	Other staff are not supportive of implementation	Setting	216 (41.5)
17	The nurse does not see the value of research for practice	Nurse	212 (40.7)
18	The research is not reported clearly and readable	Presentation	211 (40.5)
19	Statistical analyses are not understandable	Presentation	202 (38.8)
20	The nurse sees little benefits for himself or herself	Nurse	191 (36.7)
21	Implications for practice are not made clear	Presentation	189 (36.3)
22	The amount of research information is overwhelming	*	186 (35.7)
22	The research in not relevant to nurses’ practice	Presentation	186 (35.7)
23	The relevant literature is not compiled in one place	Presentation	184 (35.3)
24	The nurse feels the benefits of changing practice will be minimal	Nurse	175 (33.6)
25	The nurse is unaware of the research	Nurse	174 (33.4)
25	Manager will not allow implementation	Setting	174 (33.4)
26	The nurse is uncertain whether to believe the results of the research	Research	172 (33.0)
27	There is not a documented need to change practice	Nurse	159 (30.5)
28	The nurse in unwilling to change practice	New item	113 (21.7)
29	The nurse in unwilling to try new ideas	New item	110 (21.1)

* This item loaded a very low factor in the model identified by Funk.

### Perceptions of facilitators of research utilization

The rank order and percentage of items perceived as moderate or great facilitator are summarized in Table 3. All 8 facilitator items were perceived as moderate or great facilitator by more than half of the participants. Enhancing managerial support was ranked as the greatest facilitator (36.9%), followed by advancing education to increase research knowledge (21.1%), and increasing the time for reviewing and implementing (17.5%). Cooperation of patients to research utilization (1.0%), establishment of panel to evaluate researches (0.6%), and funding (0.4%) were identified as additional facilitators beyond the Facilitators Scale. 

**Table 3:** Facilitator items in rank order (N=521).

Rank order	Facilitator items	Rating item as moderate or great facilitator N (%)
1	Enhancing managerial support and encouraging research	455 (87.3)
2	Advancing education to increase your research knowledge base	452 (86.8)
2	Increasing the time available for reviewing and implementing	452 (86.8)
3	Improving availability and accessibility of research reports	417 (80.0)
4	Conducting more clinically focused and relevant research	411 (78.9)
5	Providing colleague support network/mechanisms	396 (76.0)
6	Improving the understandability of research reports	382 (73.3)
7	Employing nurses with research skills to serve as role models	356 (68.3)

### Factors influencing perceptions of barriers and facilitators

Results of univariate analysis are summarized in Table 4. Results of rank transformation test showed (1) participants from different hospitals had statistically significant difference in scores of the setting subscale (F=4.944, P=0.007), the presentation subscale (F=4.014, P=0.019), the BARRIERS Scale (F=3.021, P=0.050) and the Facilitators scale (F=3.382, P=0.035); (2) participants with different education background had statistically significant difference in scores of the nurse subscale (F=3.312, P=0.037) and the presentation subscale (F=3.193, P=0.042), participants with bachelor degree had significant lower scores than those with diploma degree in the presentation subscale; (3) participants with research experience had significant lower scores in the presentation subscale (F=24.999, P<0.001) and the BARRIERS Scale (F=7.024, P=0.008) than those with no research experience; (4) participants with knowledge on evidence-based nursing had lower scores in the setting subscale (F=5.170, P=0.023) and the presentation scale (F=11.678, P=0.001) than those with no evidence-based nursing knowledge. The results indicated that hospital, educational background, research experience, and knowledge on evidence-based nursing might be factors influencing perceptions of barriers and facilitators.

**Table 4:** Factors influencing perceptions of barriers and facilitators form univariate analysis (N=521).

	Hospital	Educational background	Research experience	Knowledge on EBN
Setting	F=4.944 P=0.007 C>A			F=5.170 P=0.023
Nurse		F=3.312 P=0.037		
Presentation	F=4.014 P=0.019 C>A	F=3.193 P=0.042 D>B	F=24.999 P=0.000	F=11.678 P=0.001
Barriers scale	F=3.021 P=0.050 C>A		F=7.024 P=0.008	
Facilitators scale	F=3.382 P=0.035 C>A			

Note: 1. there were three hospitals sampled in this survey. A=hospital A, C=hospital C.

2. D=diploma degree; B=bachelor degree.

3. EBN is abbreviation for Evidence-based Nursing.

Results of multivariate analysis are summarized in Table 5 (1). Hospital was a factor influencing perceptions of barriers related to the setting subscale (B= 1.331, t= 2.807, 

**Table 5:** Factors influencing perceptions of barriers and facilitators form multivariate analysis (N=521).

	Dummy variable of Hospital C	Educational background	Research experience	Knowledge on EBN
Setting	B=1.331 t=2.807 P= 0.005			B=-1.151 t=2.807 P=0.005
Nurse		B=-0.995 t=-2.048 P= 0.041		
Presentation	B=0.951 t=2.318 P= 0.021		B=-2.398 t=-4.716 P= 0.000	
Barriers scale	B=3.396 t=2.415 P= 0.016		B=-4.234 t=-2.429 P= 0.015	
Facilitators scale	B=0.925 t=2.417 P=0.016			

Note: 1. EBN is abbreviation for Evidence-based Nursing.

2. B means partial regression coefficient.

P= 0.005), the presentation subscale (B= 0.951, t= 2.318, P= 0.021), the BARRIERS Scale (B= 3.396, t= 2.415, P= 0.016) and the Facilitators Scale (B= 0.925, t= 2.417, P= 0.016), suggesting that participants from hospital C perceived more significant barriers and facilitators than those from hospital A. (2) Educational background affected perceptions of the nurse-related barriers (B= -0.995, t= -2.048, P= 0.041). It indicated that participants with higher educational background showed a more positive attitude toward research and possessed more knowledge and skills on research (3). Research experience influenced perceptions of barriers from the presentation subscale (B= -2.398, t= -4.716, P<0.001) and the BARRIERS Scale (B= -4.234, t= -2.429, P= 0.015). It revealed that nurses with research experience perceived fewer barriers in research utilization, especially in searching and understanding research (4). Knowledge on evidence-based nursing was a factor which influenced perceptions of the setting-related barriers (B= -1.151, t= 2.807, P= 0.005). It indicated that knowledge on evidence-based nursing made nurses perceive fewer barriers in working environment. 

## Discussion

### Barriers related to the setting subscale were the most influential

Setting-related barriers were found the most influential in the present study and previous barriers studies as well [11,14,19,27,31–33], possibly due to inadequate managerial support and the attributional model. Firstly, nursing science has not been fully recognized as an independent profession by managers in China and the investment in nursing is often reduced to meet the needs of medicine [34,35]. Inadequate managerial support strengthens the existing limitations of settings, such as managerial limitations to professional independence, nursing manpower shortage, decrease of nurses’ income, inadequate nursing facilities, and decrease of nursing research fund. Secondly, the attributional model proposed by Weiner suggests that people tend to attribute bad events or failures to situations i.e. external factors [36]. Actually, since the occurrence of evidence-based movement in China at the end of last century, research utilization has been gradually regarded as expectation and requirement on nurses [37,38]; however, Chinese nurses rarely implemented the research findings in their practice [39,40]. To some degree, they tend to habitually accuse the setting-related barriers for the low research utilization.

### Lack of authority, lack of time, and language barrier were identified as top three greatest barriers

Lack of authority was thought to be a major barrier in the application of research findings in nursing as it was identified as the first greatest barrier [24,31,41,42] and the second or third greatest barrier in previous researches [27,33,43–45]. Such a lack might be caused by the existing managerial system and influenced by the Chinese traditional and the nursing organizational cultures. First, existing nursing managerial system in China is a hierarchical top-down managerial system in which managers and doctors are considered as authority roles to be obeyed [39]. In such a system, nurses are not to develop working plan by themselves and their authority and independence are undermined [46]. Second, traditional values and ideas are still abode by in Chinese culture and social structure which influence ways of living and thinking in Chinese people. Among the traditional values and philosophies, Confucianism has the greatest impact on Chinese people, which appreciates the harmony with others and the respect for hierarchy. Therefore in China, to conflict with colleagues is inappropriate for educated people, and to challenge an authority or a superior role is even more inappropriate [47,48]. The reverence for authority role and superior and the obedience to situation deeply root in values, making nurses who are lower in the organizational hierarchies [49] perceive lack of authority to change practice. Third, nursing ethics regarding obedience to medical order as one of the obligations of nurses has built an organizational culture characterized by obedience [50]. Organizational culture as a kind of ideology and a kind of control function could guide the attitude and behaviour of organizational members [51]. It is hard to imagine that an obedient nurse would feel herself has enough authority to change practice.

Lack of time was also identified as the top three greatest barriers in 20 previous surveys [13]. Factors contributing to lack of time include the nurse shortage, which is the consequence of hospital authority action to reduce cost; the outdated and unreasonable national nurse-to-bed ratio standards for measuring the adequacy of staffing level in China; and nurses undertaking various non-nursing tasks [46]. Further to these factors might be that lack of time is a culturally acceptable answer in setting where busyness was valued [52].

It is understandable that language barrier was identified as the third greatest barrier, given English being not the mother tongue in China. It was listed as a major barrier in other non-English-speaking countries, such as Finland [53], Sweden [54], Greece [45], Turkey [32] and Norway [41]. Nursing science, particularly nursing research is young in China, so the outside publications in English language are more valued than domestic ones in Chinese language. Nurses in China tend to show an interest in English-language journals when evidence was needed to change practice. However, difficulty in reading English journals might have stymied many in applying nursing research findings.

The surveyed nurses identified that the lack of authority, lack of time, and language barrier were the top three greatest barriers, but in response frequency ranking, the lack of facilities, language barrier, and lack of support from physicians were the top three barriers. First, it is the rational reasoning that lack of facilities was named the most frequently perceived barriers in China, but perceivably, the surveyed nurses selected the lack of authority as the number one barrier and they also stated that the enhancement of managerial support was the top greatest facilitator. Second, differing from health system in western countries, nurses in China’s public hospitals are all full-time employed and fully loaded with various work tasks [46]. They usually work over 40 hours per week. The short-staffed and task-driven working situation might have made the surveyed nurses, to some degree, emotionally hold the lack of time as the second greatest barrier. 

### Top three greatest facilitators were enhancing managerial support, advancing education, and increasing time

The current study and a few previous studies suggest that managerial support was a key facilitator of research utilization [27,31,33]. In a practical view, managerial support could provide material support to decrease setting-related barriers, such as lack of authority, time, and facilities. In addition, according to Chinese traditional culture, the managerial support is likely to be more valued than other facilitators. Advancing education to increase research knowledge was ranked as an important facilitator in the current and previous studies [27,31,33]. Nursing education in China, especially advanced nursing education, is younger than that in the western countries. Although bachelor nursing programme was initiated in 1921, it did not flourish. Diploma programme was the only nursing education programme provided between 1949 and 1983. The re-establishment of Bachelor nursing programme took place in 1984, master nursing programme implemented in 1990, and doctoral nursing programme started in 2004. As a result, most nurses in China lack training on nursing research and evidence-based nursing [55]. The surveyed nurses stated the increasing time for reviewing and implementing to be the third greatest facilitator, since they ranked the lack of time as the second greatest barrier. The similar result was also reported in Australia [18].

### Additional barriers and facilitators listed by participants

Involvement of patients in research utilization and funding were listed as both additional barriers and facilitators in the current study, which were also reported in previous studies [9–11,15,18]. Patient involvement is required in both research utilization and evidence-based nursing; however to get patients involved, the informed consent of patients is legally required in many countries. In addition, given considerations of culture, costs, and personal habits and hobbies, patients may not give a fully compliance, which, together with the legal requirement, may account for being both barriers and facilitators in research utilization. As to funding, it is hardly possible for nurse researchers to be funded in China [56].In addition, the lack of legal protection against clinical uncertainty risk is prominent in China. Nurses usually hesitate at applying research results into practice because it is often beyond the security zone of legal regulation due to outdated practice standards. It is said that the current practice standards are usually 10 years behind the latest research results [57]. Establishing a panel to evaluate researches could tackle the deficiency in knowledge and skills on research and save individual reviewing time. Perhaps it is the reason why establishment of panel was presented as an additional facilitator. 

### Factors influencing the perceptions of barriers and facilitators

Hospital was a factor influencing perceptions of both barriers and facilitators, which may suggest that hospital as an organization/setting has multi-dimensional impact on research utilization, positively or negatively. Characteristics, such as size, location, policy, organizational culture, infrastructure, resources and interpersonal communication of an organization/setting have been associated with research utilisation in ample documents. A systematic review found that organisational factors explained 80–90% of the variance in research utilisation [58]. 

Educational background influenced the nurse-related barriers. Researchers suggested that nurses with higher academic levels usually possess a higher level need for achievement [59,60], desiring challenges [51]. Research utilization remains a challenge for many nurses in practice; therefore, nurses with a higher educational background tend to show a positive attitude towards research and research utilization as Thompson C et al. reported [5]. In China, since research curriculums are part of the bachelor nursing programme but not diploma programme, those with a bachelor degree would be more interested in and capable of conducting research utilization than those with a diploma. 

We found that research experience reduced barriers to research utilization, especially presentation-related barriers, which probably could be explained by the following reasons. Usually, conducting research results in a positive attitude toward research utilization. In addition, nurses gain necessary knowledge and skills on information retrieval, statistical analyzing and research designing through research activities, which facilitates research utilization. Moreover, research experience may help nurses obtain more managerial support needed for research utilization. Similar suggestions were also found in Royle’s work [58]. 

Knowledge on evidence-based nursing influenced setting-related barriers may be due to the fact that nurses with evidence-based knowledge usually have higher positions in hospital, which probably makes them feel less difficult in research utilization. In addition, evidence-based nursing education grants nurses the feeling of being professional authorized to change practice. Brown et al. also found that nurses with knowledge on evidence-based nursing rated lower on the setting subscale [61]. 

### Reflections on research findings

The results of this study suggest that we should formulate a comprehensive strategy to promote research utilization, taking influencing factors (hospital, educational background, research experience and knowledge on evidence-based nursing) into account, on both short- and long-term levels. Since the generally existing lack of knowledge and skills on research and on evidence-based nursing, serious shortage of staff, organizational culture, and most importantly, current managing system and traditionally-hold values, can not be changed effectively in a short period of time in China. Therefore establishing panels to publish guidelines with clear application notes and update it regularly and requiring the implement of the published guidelines as practicing standard officially may be a practical solution to promote research utilization promptly in China. 

From a long-term and broader perspective, the following approaches probably should be also considered. First of all, managerial support should be improved. Specifically speaking, authorization of nurse to change practice, creation of a kind of “ready to change” organizational culture, and multi-disciplinary collaboration are needed to put into agenda. Secondly, education on research knowledge and skills should be advanced to improve individual ability of applying research findings. Perhaps education of leadership in practice should be considered, too. These approaches would fundamentally boost research utilization. 

Given Chinese traditional culture, existing health system and educational development level, no matter from the short-term or long-term perspective, enhancing managerial support to research utilization might be the most important approach.

While our findings remain a great degree of consistency with findings from other barriers studies over time and across geographic settings, many new changes have occurred. For example, advances of nursing education and information technology, and development of healthcare professional environment. This phenomenon prompts us to think over a series of questions. What is under this consistency phenomenon? Are those advances and developments sufficient to change nurses’ perceptions, or in other word, to eliminate the real barriers? What is the core of solutions to push research use forward? With limited successful experience to refer to, we still have a long way to go to promote research utilization. 

### Limitations

This study was conducted at three tertiary-level hospitals in a limited sample and thus has a limited power to be generalized to reflect the entire situation of research utilization in China. The self-rated research method might not be robust enough to objectively present what the surveyed nurses exactly thought over the research utilization. Therefore, further studies with qualitative methods such as observation or depth interview are needed.

## Conclusions

Research utilization has not been widely implemented yet in China due to various barriers. The setting-related barriers were most influential, with the lack of authority, language barrier and the lack of time being the top three greatest barriers. Enhancing managerial support, advancing education to increase research knowledge, and increasing the time for reviewing and implementing were the greatest facilitators. Hospital, educational level, research experience and knowledge on evidence-based nursing were influencing factors to research utilization. A comprehensive strategy should be formulated to improve research utilization. Enhancing managerial support might be the most promising facilitator, given Chinese traditional culture and current health care system. 

Since the involvement of patients in research utilization and funding were repeatedly identified as influencing factors in research utilization and both are important in current nursing practice, especially the involvement of patients, so it is reasonable to add them into the BARRIERS Scale to improve its validity.
